# Supplementary material for: User Experiences and Attitudes Toward Sharing Wearable Activity Tracker Data with Healthcare Providers: A Cross-Sectional Study
Source: Healthcare (Basel). 2025 May 22;13(11):1215. doi: 10.3390/healthcare13111215 (PMC12154086; doi:10.3390/healthcare13111215)
Supplement: Supplementary file 1 [file healthcare-13-01215-s001.zip › Supplementary File S1 Survey Instrument.pdf]

Supplementary file 1. Survey Instrument

Do you use, or have you previously used a wearable activity tracker (WAT) (Apple watch, Fitbit, Garmin etc?). A WAT can be defined as any wearable device which objectively measures physical activity levels and provides feedback.

You are eligible to take part in this survey if you:

- Are over the age of 18
- Currently use or have previously used a WAT within the last 3 years and for at least 1 month
- Have a WAT that measures daily physical activity metrics
- Have a WAT that interacts with an associated app/software on a smartphone or computer

I acknowledge that by completing and submitting the survey I agree for my anonymous data to be used as part of the research.

☐ Yes
☐ No

Thank you for choosing to take part in this survey!

Question	Response Option/s
**SOME BASIC DETAILS ABOUT YOU**	
Sex	• Male<br>• Female<br>• Non-binary<br>• Prefer not to say<br>• Other (please specify)
How old are you?	Open field, numerical
What country do you live in?	Drop down menu
What is the highest level of education you have completed?	• Primary School<br>• High school – Year 12 or equivalent<br>• High school – Year 11 or equivalent<br>• High school – Year 10 or equivalent<br>• [Vocational qualification/trade school/community college/certificate/diploma/apprenticeship]<br>• University bachelor's degree<br>• Post graduate degree (E.g., Master's Degree)

**HOW YOU USE YOUR WEARABLE ACTIVITY TRACKER**	
Are you currently using a wearable activity tracker?	• Yes, I am currently using a wearable activity tracker<br>• No, I previously used a wearable activity tracker<br>• I have never used a wearable activity tracker (If answered, end survey)
How did you get your wearable activity tracker?	• I bought it for myself<br>• Gift from friend/family<br>• Provided by health insurer<br>• Provided by workplace<br>• Other (please specify)
Before you got your wearable activity tracker, in a typical week, on how many days did you get at least 30 minutes of physical activity?	Drop down menu (0-7)
In the last week, how many days did you get at least 30 minutes of physical activity?	Drop down menu (0-7)
**FORMER USERS ONLY**	
What is the reason you stopped using your wearable activity tracker? Tick all that apply	• It broke<br>• I wasn't using it enough<br>• Got lost<br>• It was difficult to understand<br>• Technical difficulties<br>• It wasn't helping with my goals<br>• I didn't like it<br>• I found it intrusive<br>• I was experiencing negative psychological impacts<br>• I learnt everything I could from it<br>• Other (please specify)
Which wearable activity tracker did you use? Please specify the brand and model, if possible	Open field, free text
Did you have any complaints with your wearable activity tracker? Tick all that apply	• None<br>• Technical issues<br>• It fell off<br>• It didn't fit<br>• It was uncomfortable<br>• Lost it<br>• Low battery life<br>• General wear and tear<br>• It often didn't match my outfit<br>• Problems with the screen<br>• Problems uploading the data to supporting software<br>• Problems interpreting the data<br>• Problems with navigation of supporting website/app/technology<br>• Inaccurate at recording data

	• Other (please specify)
Overall, I had a positive experience using my wearable activity tracker	• Disagree strongly<br>• Somewhat disagree<br>• Neutral<br>• Somewhat agree<br>• Agree strongly
How long did you use your wearable activity tracker?	• 0-3 months<br>• >3-6 months<br>• >6-12 months<br>• >1-2 years<br>• >3-5 years<br>• >6-8 years<br>• >9+ years
What was your main motivation for using your wearable activity tracker? (Tick all that apply)	• To improve fitness<br>• To improve health<br>• To improve appearance<br>• To monitor activities<br>• To share my activity<br>• To compete with family/friends<br>• To keep up with new technology<br>• Other (please specify)
Which graph best matched how your activity levels changed after getting your wearable activity tracker?<br>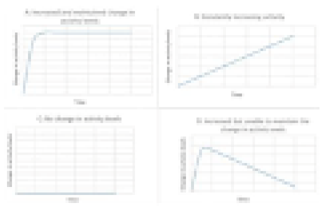	• A<br>• B<br>• C<br>• D<br>• Other (please describe)
How much do you agree/disagree with the following statements? When I had a wearable activity tracker I…<br>• Ate healthier<br>• Incorporated more physical activity in my day<br>• Slept more	• Disagree strongly<br>• Somewhat disagree<br>• Neutral<br>• Somewhat agree<br>• Agree strongly
How much do you agree/disagree with the following statements? I found the monitoring on my wearable activity tracker useful…<br>• Real time (i.e., tracking activities on that day)<br>• Long term (i.e., tracking change or maintenance of activity across days	• Disagree strongly<br>• Somewhat disagree<br>• Neutral<br>• Somewhat agree<br>• Agree strongly
How much do you agree/disagree with the following statement? I would be inclined to use a wearable activity tracker again?	• Disagree strongly<br>• Somewhat disagree<br>• Neutral

	• Somewhat agree<br>• Agree strongly
What social networks did you share your activity data in? Tick all that apply	• Facebook<br>• Twitter<br>• Instagram<br>• Sharing feature within the wearable's software<br>• I didn't share my data<br>• Other (please specify)
(If answered yes to Q27) Why did you share your activity data on social networks? Tick all that apply	• To share progress<br>• To compete with friends<br>• To get encouragement from others<br>• To motivate others<br>• I didn't share my data<br>• Other (please specify)
Did you ever consciously cheat to record your activity on your wearable activity tracker?	• Yes (If so, how?)<br>• No
**HOW YOUR WEARABLE ACTIVITY TRACKER MAKES YOU FEEL**	
How much do you agree/disagree with the following statements? When I used my wearable activity tracker, it made me feel…<br>• Guilty<br>• Empowered<br>• Motivated<br>• Accountable<br>• Self-conscious<br>• Anxious	• Disagree strongly<br>• Somewhat disagree<br>• Neutral<br>• Somewhat agree<br>• Agree strongly
How much do you agree/disagree with the following statements? When I didn't use/forgot/couldn't use my wearable activity tracker, it made me feel…<br>• Guilty<br>• Frustrated<br>• Anxious<br>• Liberated	• Disagree strongly<br>• Somewhat disagree<br>• Neutral<br>• Somewhat agree<br>• Agree strongly
Here are a number of personality traits that may or may not apply to you. Please indicate the extent to which you agree/disagree with each pair. You should rate the extent to which the pair of traits applies to you, even if one characteristic applies more strongly than the other.<br>• Extroverted, enthusiastic<br>• Critical, quarrelsome<br>• Dependable, self-disciplined<br>• Anxious, easily upset<br>• Open to new experiences, complex<br>• Reserved, quiet	• Disagree strongly<br>• Disagree moderately<br>• Disagree a little<br>• Neutral<br>• Agree a little<br>• Agree moderately<br>• Agree strongly

• Sympathetic, warm<br>• Disorganised, careless<br>• Calm, emotionally stable<br>• Conventional, uncreative	
Do you have any other comments you wish to share?	• Yes (If so, please specify)<br>• No
**CURRENT USERS ONLY**	
Which wearable activity tracker do you use? Please specify the brand and model, if possible	Open field, free text
Have you ever had another wearable activity tracker? If so, how many?	• Yes<br>- Drop down menu (1-10)<br>• No
How frequently do you use each of these wearable activity tracker features?<br>• Active minutes<br>• Steps<br>• Stairs<br>• Sleep<br>• Heart rate<br>• Energy burned<br>• Energy consumed (food)<br>• Meditation<br>• Workouts<br>• Receiving calls/text messages<br>• Contactless pay<br>• Calendar reminders<br>• Connection to other devices (e.g., computer, phone, tablet)<br>• Sharing data (e.g., on social media)<br>• Other (please specify)	• Never<br>• Rarely (e.g. once a month or less)<br>• Sometimes (e.g. once a week)<br>• Frequently (e.g. most days)<br>• Very frequently (e.g. multiple times a day)
How much do you agree/disagree with the following statements? I find the following features on my wearable activity tracker useful…<br>• Active minutes<br>• Steps<br>• Stairs<br>• Sleep<br>• Heart rate<br>• Energy burned<br>• Energy consumed (food)<br>• Meditation<br>• Workouts<br>• Receiving calls/text messages<br>• Contactless pay<br>• Calendar reminders<br>• Connection to other devices (e.g., computer, phone, tablet)<br>• Sharing data (e.g., on social media)	• Disagree strongly<br>• Somewhat disagree<br>• Neutral<br>• Somewhat agree<br>• Agree strongly

• Other (please specify)	
Following full charge, how long does your wearable activity trackers battery last?	Drop down menus: Hours/days/weeks/months
Do you have any complaints with your wearable activity tracker? Tick all that apply	• None<br>• Technical issues<br>• It falls off<br>• It doesn't fit<br>• It is uncomfortable<br>• Lost it<br>• Low battery life<br>• General wear and tear<br>• It often doesn't match my outfit<br>• Problems with the screen<br>• Problems uploading the data to supporting software<br>• Problems interpreting the data<br>• Problems with navigation of supporting website and technology<br>• Inaccurate at recording data<br>• Other (please specify)
What would you change about your wearable activity tracker?	• Longer battery life<br>• More aesthetic<br>• Easier to use<br>• More features (please specify)<br>• Other (please specify)
How often do you use your wearable activity tracker?	• Every day including overnight<br>• Every day during waking hours<br>• Once a week<br>• When I remember<br>• Other (please specify)
How long have you used your current wearable activity tracker for?	• 0-3 months<br>• >3-6 months<br>• >6-12 months<br>• >1-2 years<br>• >3-5 years<br>• >6-8 years<br>• >9+ years
How long have you used any wearable activity tracker for?	• 0-3 months<br>• >3-6 months<br>• >6-12 months<br>• >1-2 years<br>• >3-5 years<br>• >6-8 years<br>• >9+ years
How long are you planning to continue using your wearable activity tracker?	• 0-3 months<br>• >3-6 months<br>• >6-12 months<br>• >1-2 years

	• >3-5 years<br>• >6-8 years<br>• >9+ years<br>• Indefinitely
Overall, I have had a positive experience using my wearable activity tracker	• Disagree strongly<br>• Somewhat disagree<br>• Neutral<br>• Somewhat agree<br>• Agree strongly
What is your main motivation for using your wearable activity tracker?	• To improve fitness<br>• To improve health<br>• To improve appearance<br>• To monitor activities<br>• To share my activity<br>• To compete with family/friends<br>• To keep up with new technology<br>• Other (please specify)
Do you use your wearable activity tracker for a daily health goal?	• Yes<br>• No
Please specify your daily goal e.g.<br>• Active minute goal<br>• Step count goal<br>• Sleep goal<br>• Energy burned goal<br>• Energy consumed (food) goal<br>• Meditation goal<br>• Workout goal<br>• Other	Open field, free text
Which graph best matches how your activity levels have changed since getting your wearable activity tracker?<br>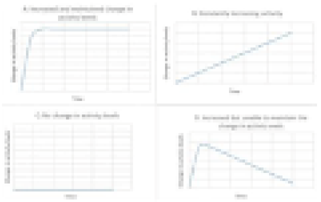	• A<br>• B<br>• C<br>• D<br>• Other (please describe)
How much do you agree/disagree with the following statements? Since using my wearable activity tracker, I now...<br>• Eat healthier<br>• Incorporate more activity in my day<br>• Sleep more	• Disagree strongly<br>• Somewhat disagree<br>• Neutral<br>• Somewhat agree<br>• Agree strongly
Which device(s) do you use with your wearable activity tracker? Tick all that apply	• Smartphone<br>• Tablet<br>• Computer/laptop<br>• None
What is the main device you use to track your activity?	• Device itself<br>• Smartphone

	• Tablet<br>• Computer/laptop<br>• None
What social networks do you share your data in? Tick all that apply	• Facebook<br>• Twitter<br>• Instagram<br>• With the wearables software<br>• I don't share my activity data<br>• Other (please specify)
Why do you share your activity on social networks? Tick all that apply	• To share progress<br>• To compete with friends<br>• To get encouragement from others<br>• To motivate others<br>• I don't share data<br>• Other (please specify)
How many times a day do you check your progress?	Open field, numerical
How much do you agree/disagree with the following statements? I find the monitoring on my wearable activity tracker useful…<br>• Real time (i.e. tracking activities on that day)<br>• Long term (i.e., tracking change or maintenance of activity across days	• Disagree strongly<br>• Somewhat disagree<br>• Neutral<br>• Somewhat agree<br>• Agree strongly
How much do you agree/disagree with the following statements? When I'm using my wearable activity tracker, it makes me feel…<br>• Guilty<br>• Empowered<br>• Motivated<br>• Accountable<br>• Self-conscious<br>• anxious	• Disagree strongly<br>• Somewhat disagree<br>• Neutral<br>• Somewhat agree<br>• Agree strongly
How much do you agree/disagree with the following statements? When I'm not using/forget/can't use my wearable activity tracker, it makes me feel…<br>• Guilty<br>• Frustrated<br>• Anxious<br>• Liberated	• Disagree strongly<br>• Somewhat disagree<br>• Neutral<br>• Somewhat agree<br>• Agree strongly
Have you consciously cheated to record your activity on your wearable activity tracker?	• Yes (If so, how?)<br>• No
What social networks do you share your data in? Tick all that apply	• Facebook<br>• Twitter<br>• Instagram<br>• With the wearables software<br>• I don't share my activity data<br>• Other (please specify)

Why do you share your activity on social networks? Tick all that apply	• To share progress<br>• To compete with friends<br>• To get encouragement from others<br>• To motivate others<br>• I don't share data<br>• Other (please specify)
Here are a number of personality traits that may or may not apply to you. Please indicate the extent to which you agree/disagree with each pair. You should rate the extent to which the pair of traits applies to you, even if one characteristic applies more strongly than the other.<br>• Extroverted, enthusiastic<br>• Critical, quarrelsome<br>• Dependable, self-disciplined<br>• Anxious, easily upset<br>• Open to new experiences, complex<br>• Reserved, quiet<br>• Sympathetic, warm<br>• Disorganised, careless<br>• Calm, emotionally stable<br>• Conventional, uncreative	• Disagree strongly<br>• Disagree moderately<br>• Disagree a little<br>• Neutral<br>• Agree a little<br>• Agree moderately<br>• Agree strongly
Do you have any other comments on your experience with wearable activity trackers you wish to share?	• Yes (If so, please specify)<br>• No

Thank you very much for completing this survey! If you would like to enter the draw to win 1x $100 Coles/Myer gift voucher, please provide your email address:
