# Supplementary material for: User Experiences and Attitudes Toward Sharing Wearable Activity Tracker Data with Healthcare Providers: A Cross-Sectional Study
Source: Healthcare (Basel). 2025 May 22;13(11):1215. doi: 10.3390/healthcare13111215 (PMC12154086; doi:10.3390/healthcare13111215)
Supplement: Supplementary file 1 [file healthcare-13-01215-s001.zip › Supplementary File S2 List of Countries.pdf]

List of Countries	Count
Afghanistan	3
Albania	5
Algeria	2
Andorra	2
Angola	1
Antigua and Barbuda	3
Argentina	4
Armenia	6
Australia	122
Austria	2
Barbados	1
Belgium	2
Belize	1
Bolivia	1
Brazil	1
Cameroon	1
China	2
Colombia	1
Cyprus	1
Denmark	1
Eritrea	1
Gambia	1
Germany	1
India	5
Lithuania	1
New Zealand	1
Sri Lanka	1
Suriname	1
Tuvalu	1
Uganda	2
United Arab Emirates	1
United Kingdom of Great Britain and Northern Ireland	1
United States of America	268
