# Supplementary material for: User Experiences and Attitudes Toward Sharing Wearable Activity Tracker Data with Healthcare Providers: A Cross-Sectional Study
Source: Healthcare (Basel). 2025 May 22;13(11):1215. doi: 10.3390/healthcare13111215 (PMC12154086; doi:10.3390/healthcare13111215)
Supplement: Supplementary file 1 [file healthcare-13-01215-s001.zip › Supplementary File S3 (Tables S1-S4).pdf]

Supplementary File S3

Supplementary Table S1: Willingness

	**Coefficient**	**Std. err.**	**z**	**P>z**	**[95% conf. interval]**	
**Gender (constant = male)**						
Female	0.96	0.50	1.94	0.052	-0.01	1.94
**Age (constant = 18-24)**						
25-34	-0.92	0.81	-1.14	0.256	-2.51	0.67
35-44	-1.20	0.84	-1.44	0.150	-2.84	0.44
45-54	-0.40	1.07	-0.37	0.710	-2.50	1.70
55+	-0.78	1.33	-0.59	0.558	-3.37	1.82
**Country of origin (constant = other)**						
Australia	1.17	0.70	1.67	0.095	-0.20	2.55
United States	0.63	0.52	1.22	0.222	-0.38	1.65
**Education (constant = secondary school or below)**						
Cert/Dip	-0.19	0.74	-0.26	0.796	-1.64	1.26
University bachelor's	0.28	0.51	0.55	0.580	-0.71	1.27
Post grad	-0.41	0.64	-0.64	0.522	-1.66	0.84
**Self-rated health (constant = excellent)**						
Very good	0.33	0.53	0.61	0.541	-0.72	1.37
Good	0.27	0.57	0.47	0.640	-0.86	1.39
Poor-fair	0.00	(empty)				
**Chronic conditions (constant = none)**						
1	-0.10	0.48	-0.22	0.827	-1.04	0.83
2 or more	0.32	0.58	0.56	0.577	-0.81	1.45
**Constant**	2.22	1.00	2.23	0.026	0.27	4.17
Logistic regression	Number of observations	=	413			
	LR chi2(14)	=	13.58			
	Prob > chi2	=	0.4818			
Log likelihood = -98.19772	Pseudo R2	=	0.0647			

Supplementary Table S2: Discussed

	Coefficient	Std. err.	z	P>z	[95% conf. interval]	
**Gender (constant = male)**						
Female	-0.37	0.22	-1.72	0.086	-0.80	0.05
**Age (constant = 18-24 years)**						
25-34	0.33	0.33	1.01	0.311	-0.31	0.98
35-44	0.50	0.35	1.41	0.158	-0.19	1.20
45-54	0.52	0.42	1.23	0.218	-0.31	1.35
55+	0.17	0.59	0.29	0.770	-0.98	1.33
**Country of origin (constant = other)**						
Australia	-0.83	0.36	-2.28	0.022	-1.54	-0.12
United States	0.07	0.32	0.20	0.838	-0.56	0.69
**Education (constant = secondary school or below)**						
Cert/Dip	0.10	0.39	0.26	0.798	-0.67	0.87
University bachelor's	0.51	0.26	1.98	0.047	0.01	1.01
Post grad	0.35	0.36	0.98	0.326	-0.35	1.06
**Self-rated health (constant = excellent)**						
Very good	-0.02	0.30	-0.05	0.956	-0.59	0.56
Good	0.38	0.31	1.20	0.229	-0.24	0.99
Poor-fair	0.94	0.48	1.93	0.053	-0.01	1.88
**Chronic conditions (constant = none)**						
1	0.55	0.25	2.23	0.026	0.07	1.04
2 or more	0.84	0.28	3.00	0.003	0.29	1.39
**Constant**	-1.11	0.50	-2.21	0.027	-2.10	-0.13
Number of observations	446					
LR chi2(15)	51.21					
Prob > chi2	<0.001					
Pseudo R2	0.083					
Log likelihood	-282.53					

Supplementary Table S3: Shared

	Coefficient	Std. err.	z	P>z	[95% conf. interval]	
**Gender (constant = male)**						
Female	-0.70	0.22	-3.13	0.002	-1.14	-0.26
**Age (constant = 18-24)**						
25-34	0.45	0.35	1.31	0.191	-0.23	1.13
35-44	0.94	0.37	2.51	0.012	0.21	1.66
45-54	0.70	0.44	1.60	0.109	-0.16	1.57
55+	0.35	0.61	0.57	0.568	-0.84	1.54
**Country of origin (constant = other)**						
Australia	-0.99	0.37	-2.66	0.008	-1.72	-0.26
United States	-0.02	0.32	-0.08	0.939	-0.65	0.60
**Education (constant = high school or below)**						
Cert/Dip	0.29	0.40	0.73	0.463	-0.49	1.08
University bachelor's	0.45	0.26	1.73	0.084	-0.06	0.96
Post grad	0.20	0.37	0.53	0.594	-0.53	0.93
**Self-rated health (constant = excellent)**						
Very good	-0.36	0.30	-1.22	0.223	-0.95	0.22
Good	-0.02	0.31	-0.07	0.941	-0.64	0.59
Poor-fair	0.47	0.48	0.99	0.320	-0.46	1.41
**Chronic conditions (constant = none)**						
1	0.59	0.26	2.32	0.021	0.09	1.09
2 or more	0.66	0.29	2.32	0.020	0.10	1.22
**Constant**	-0.91	0.51	-1.78	0.075	-1.91	0.09
Number of observations	446					
LR chi2(15)	57.71					
Prob > chi2	<0.001					
Pseudo R2	0.095					
Log likelihood	-275.08					

Supplementary Table S4: Concerns

	Coefficient	Std. err.	z	P>z	[95% conf. interval]	
**Gender (constant = male)**						
Female	-0.46	0.26	-1.80	0.072	-0.97	0.04
**Age (constant = 18-24)**						
25-34	0.35	0.41	0.85	0.395	-0.45	1.15
35-44	0.69	0.43	1.60	0.111	-0.16	1.55
45-54	1.23	0.50	2.47	0.013	0.25	2.20
55+	0.09	0.77	0.12	0.908	-1.42	1.59
**Country of origin (constant = other)**						
Australia	-1.78	0.43	-4.17	0.000	-2.62	-0.94
United States	-1.01	0.34	-3.01	0.003	-1.68	-0.35
**Education (constant = high school or below)**						
Cert/Dip	-0.47	0.47	-1.02	0.309	-1.39	0.44
University bachelor's	-0.29	0.28	-1.01	0.312	-0.84	0.27
Post grad	-0.29	0.42	-0.69	0.488	-1.12	0.53
**Self-rated health (constant = excellent)**						
Very good	-0.12	0.35	-0.34	0.734	-0.80	0.56
Good	-0.05	0.37	-0.15	0.883	-0.77	0.66
Poor-fair	0.14	0.53	0.26	0.793	-0.89	1.17
**Chronic conditions (constant = none)**						
1	0.83	0.31	2.63	0.008	0.21	1.44
2 or more	1.09	0.34	3.25	0.001	0.43	1.75
Constant	-0.76	0.57	-1.34	0.181	-1.88	0.36
Number of observations	446					
LR chi2(15)	57.01					
Prob > chi2	<0.001					
Pseudo R2	0.112					
Log likelihood	-227.12					
